# Toy exploration in gifted word learner dogs and typical dogs

**DOI:** 10.1007/s10071-026-02047-3

**Published:** 2026-01-29

**Authors:** Andrea Sommese, Ádám Miklósi, Silvia Nostri, Andrea Temesi, Claudia Fugazza

**Affiliations:** 1 Department of Ethology, Eötvös Loránd University, Pázmány P. s 1c, 6th Floor, Budapest, 1117 Hungary; 2 MTA-ELTE Comparative Ethology Research Group, Budapest, Hungary; 3 Department of Life Sciences and Systems Biology, University of Turin, Via Accademia Albertina 13, 10125 Torino, Italy; 4 ELTE NAP Comparative Ethology Research Group, Budapest, Hungary; 5 Comparative Cognition, Messerli Research Institute, University of Veterinary Medicine Vienna, Medical University of Vienna, Veterinärplatz 1, 1210 Vienna, Austria; 6 Centre for Animal Nutrition and Welfare, Clinical Department for Farm Animals and Food System Science, University of Veterinary Medicine Vienna, Vienna, Austria

**Keywords:** Gifted word learner dog, Object exploration, Object discrimination, Social interaction, Neophilia

## Abstract

Labelling has a pronounced effect on increasing infants’ attention to objects. At the same time, infants actively seek social cues when presented with novel objects and early signs of communicative intent are considered essential for language learning. Although no other species has been shown to possess language in its integrity, a small group of rare individual dogs (Gifted Word Learners, GWL) shows a limited subset of language-related skills: the capacity to form an extensive vocabulary of object verbal labels rapidly. Comparing these dogs to typical dogs that lack this capacity provides a unique opportunity to study the relationship between vocabulary acquisition and other cognitive traits in a non-human, non-linguistic species that evolved and developed in the human environment. The present study compares the object preferences and tendency to seek social interactions of GWL (*N* = 10) and typical (*N* = 21) dogs. During a two-week familiarisation phase, the caretakers and the dogs engaged in play with four dog toys, only two of which were labelled. In contrast, the other two were not labelled during the playful interaction. The subsequent test phase consisted of two trials in which these four toys, along with two novel ones, were placed on the floor, and the caretakers remained passive. The dogs were given 90 s to explore freely. The results did not provide evidence for significant differences between GWL dogs and T dogs’ exploration of the labelled, unlabelled and novel objects. GWL dogs, however, demonstrated a significantly higher propensity to interact with their caretakers while holding a toy in their mouths, notably, mainly presenting the novel toy to their caretakers. GWL dogs’ tendency to interact with the passive caretaker may suggest a greater interest in the social aspect of interacting with objects.

## Introduction

Dogs are a popular model for investigating social cognition (e.g., Aria et al. 2021), thanks to their evolutionary and developmental history in the human environment (Miklosi 2014). While clearly dogs do not acquire human language, a group of rare individual dogs have demonstrated the capacity to rapidly learn a vast vocabulary of verbal labels associated with objects during natural interactions in human families (Dror et al. 2023; Griebel and Oller 2012; Kaminski et al. 2004, 2009; Miklosi 2014; Pilley and Reid 2011). Most dogs do not exhibit this capacity (Fugazza et al. 2021; Ramos and Mills 2019), these exceptional individuals were labelled Gifted Word-Learner (hereafter GWL) dogs (Dror et al. 2023; Fugazza et al. 2021), with the use of the word “Word” in the narrow sense of verbal label referringto objects. When comparing GWL dogs to their typical (T) counterparts, the distinctions observed so far were twofold: only GWL dogs exhibited proficiency in acquiring a vocabulary of object labels. They were reported to present an increased inclination towards playfulness (Fugazza et al. 2022).

Family dogs and infants develop in a relatively similar linguistic environment: the human family. Unlike other non-human species studied in relation to language, pet dogs develop in a human household as their species-typical environment, meaning that daily exposure to human speech is part of their natural developmental ecology rather than an experimental or training context. Moreover, GWL dogs learn verbal labels during naturally occurring interactions without their caretakers explicitly intending to teach or train them (Dror et al. 2023). However, it has to be maintained that there are also several differences in the way human infants and dogs (or other species) are exposed to language (see Pepperberg 2024). Studying the similarities and differences between dogs with a vocabulary of object labels and T dogs without the ability to acquire it may shed some light on how vocabulary acquisition influences other cognitive processes, such as attentional biases (Hauser et al. 2002; LaTourrette et al. 2023; Pomper and Saffran 2019). Moreover, in infants, learning words is essentially a social skill (Degotardi 2017; Tomasello 2000), but it is currently not known whether this is the case for GWL dogs as well. In this framework, GWL dogs offer a unique comparative model for examining attentional biases linked to word learning in a non-human species, and for beginning to explore how verbal label learning may relate to social motivation in individuals with vocabulary-learning abilities.

Advanced social cognition is necessary for children to acquire language (e.g., Carpenter et al. 1998; Tomasello 2003). Early signs of communicative intent and shared attention are considered essential for language learning (Degotardi 2017; Tomasello 2000). From around 8 to 9 months, infants start to use pointing and gaze alternation between objects and caregivers to share attention, and these are considered a foundational sign of communicative intent (e.g., Carpenter et al. 1998). Labelling, the act of assigning vocal labels to objects, is a fundamental aspect of language acquisition and has a profound effect on infants’ attention to their environment (LaTourrette et al. 2023). Previous findings have shown that labelling can direct infants’ attention towards an object and enhance their attention to that object, even beyond the initial period of labelling (Baldwin and Markman 1989). Furthermore, the lexical status of an image can affect infants’ behaviour and orientation (Schafer et al. 1999).

Object exploration is essential for cognitive development. It is widespread in human and non-human animals and is thought to confer benefits by allowing individuals to develop motor skills and engage in novel behaviours. For example, a few studies suggest that active object exploration plays a significant role in children’s language development and global cognitive skills (e.g., Ruff et al. 1984; Zuccarini et al. 2017). In non-human animals, object exploration can, for instance, lead to innovative foraging strategies (e.g., Auersperg 2015; Bateson and Martin 2013), and dogs also prefer novel objects over familiar ones (Kaulfuß and Mills 2008; Kniowski 2012).

We conducted a study to examine the relationship between the skill of learning object labels and dogs’ tendency to interact with labelled, unlabelled and novel objects and with the caretaker. For this purpose, we adapted and integrated the one-trial Novel Object Recognition test, which has been successfully used to measure the difference in the exploration time of novel and familiar objects in rats, mice, birds, reptiles, and fish (e.g., Antunes and Biala 2012; Blaser and Heyser 2015). Whilst our original hypothesis was that both T and GWL dogs would show curiosity towards novel objects (Kaulfuß and Mills 2008; Kniowski 2012), we hypothesised that GWL dogs might show an increased preference for the novel objects, as this may aid them in learning the association between a label and the corresponding object. Because labelling directs infants’ attention to the labelled objects (Baldwin and Markman 1989; Schafer et al. 1999), and since only GWL dogs acquire object labels, we hypothesised that GWL dogs would show more interest in labelled objects and less interest in unlabelled objects than T dogs. In addition, as GWL dogs are reported to be more playful (Fugazza et al. 2022) and vocabulary acquisition in GWL dogs takes place during social-playful interactions with their caretakers (Dror et al. 2023; Fugazza and Miklósi 2020), we expected that GWL dogs would tend to interact with the caretaker more than T dogs during the experiment.

## Methods

### Ethics statement

The study has complied with all relevant ethical regulations for animal testing. Ethical approval for this study was obtained from the National Scientific Ethical Committee on Animal Experimentation (PEI/001/1057-6/2015). The caretakers volunteered with their dogs to participate in the study and gave written informed consent.

### Subjects

We recruited *N* = 10 GWL dogs, all Border Collies (5 males, 5 females; mean age 6.4 ± 3.2 years), and *N* = 21 T Border Collies (10 males, 11 females; mean age 3.4 ± 1.6). As there is a preponderance of Border collies among the GWL dogs (Dror et al. 2023), we only recruited dogs of this breed. The GWL dogs included in this study were previously tested for their acquired vocabulary (Dror et al. 2023). Because the ability to learn even only two object labels is extremely rare in dogs (Fugazza et al. 2021; Ramos and Mills 2019), we assumed that the T dogs recruited would not learn the toy labels during the experiment. However, to ensure that this was the case, we also conducted a Vocabulary Assessment Test (VAT, see below) to test the dogs’ acquisition of the labels used in the study. Two T dogs were excluded because their caretakers discontinued participation after the initial familiarisation phase. All GWL dogs had been shown to know the names of more than 20 dog toys in previous studies (Dror et al. 2023; Fugazza et al. 2021). Since GWL dogs’ vocabulary is constituted of toy labels and learning occurs during playing with toys, we only recruited subjects (both T and GWL) that were motivated to interact with toys, as reported by their caretakers. To be included in the study, dogs had to be at least eight months old, healthy, and without a history of behavioural problems.

### Toys

**Fig. 1:**
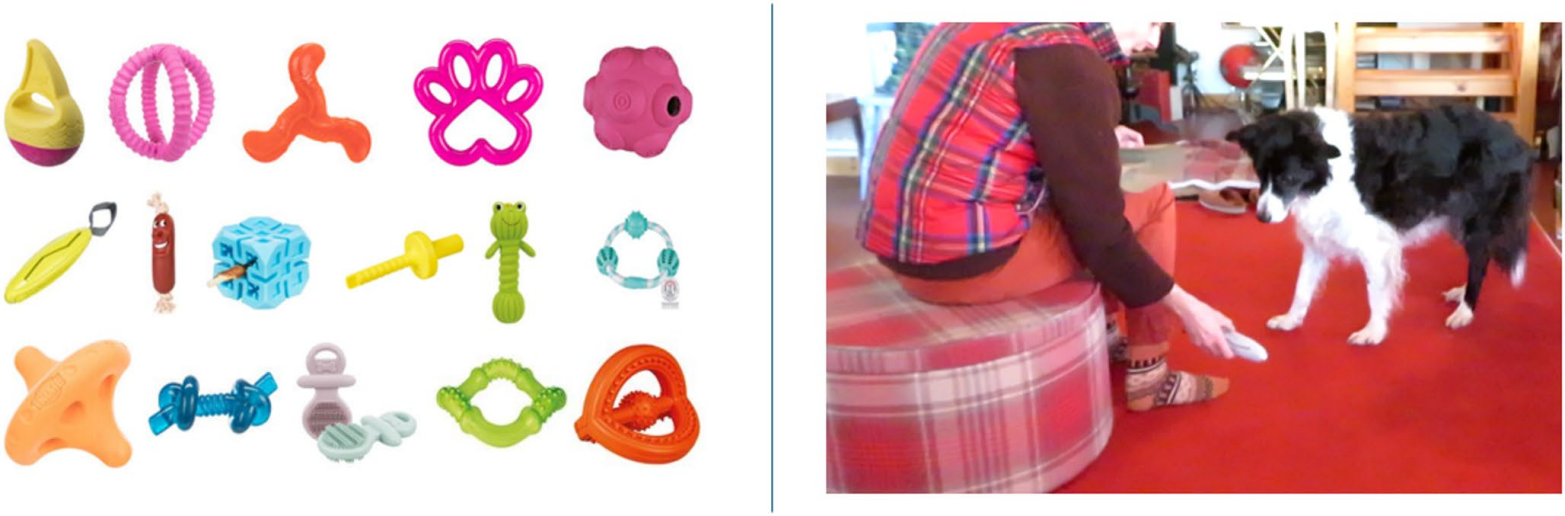
The figure shows the different toys used during the experiment. On the right, one of the GWL dogs (Roy) is playing with his caretaker

A list of 15 toys (Fig. 1) was discussed with the participants to ensure that none of the dogs, GWL or T, had experienced a similar toy before this experiment. TRIXIE© provided all toys. A selection of 8 toys was semi-randomly chosen (to ensure the novelty of all toys for each dog) and sent to each participant.

### Procedure

Before the tests, the dogs received a two-week familiarisation period (see below). The 8 toys received by each caretaker were randomly assigned to the following categories: two labelled, two unlabelled, and four novel toys (two novel A and two novel B). The assignment of the toys to the categories was randomised for each dog. After the familiarisation period, testing was conducted remotely using an online software (i.e., Streamyard). The dogs were tested in their homes, with their caretakers present to assist with the testing and live instruction from the experimenters (AS and AT) through the online streaming platform.

### Familiarisation

During the two-week familiarisation phase, the caretaker and the dog played with four toys (two labelled and two unlabelled) at least once a day for approximately 10 min. The experimenter was connected online with the caretaker for at least 1–2 initial sessions. Each toy was presented separately, and this phase followed a specific schedule, with labelled and unlabelled toys introduced on different days. The order of introduction of the toys was constant across dogs (although which toy was assigned to which category was different across dogs). The familiarisation was carried out as follows:

**Table:** 

Familiarisation schedule	
Day 1–3	Introduce labelled toy 1
Day 4–6	Introduce unlabelled toy 1
Day 7–9	Introduce labelled toy 2
Day 10–12	Introduce unlabelled toy 2
Day 12–15	Play with all the toys

When playing with labelled toys, the caretaker crouched on the floor and moved the toy, dragging it on the floor to stimulate the dog’s interest. The caretaker repeatedly said the name of the toy (e.g., “Hey! Look at the < toy name>, here is the < toy name>!“). After the dog picked up the toy, the caretaker played with it briefly by pulling it, praising the dog for taking the toy and repeating the name of the toy (e.g., “Good dog, this is the < toy name>!“). When playing with unlabelled toys, the caretaker played in the same way (e.g., uttering “Let’s play!” or “Come get it”), still praising the dog but never saying the name of the toy. Once a toy (labelled or unlabelled) was introduced, it always remained available to the dog. The caretakers were asked to devote an equal amount of play time to all the toys and to play with the toys belonging to the two categories for the same amount of time.

### Testing

On day 15, the dogs participated in a test, which consisted of two trials of 90 s each. The test was conducted in a room with no distractors, such as other toys or food, in the household, in the presence of the caretaker, who was instructed to remain passive and ignore the dog. Overall, 8 toys were used in the test: the two labelled toys, the two unlabelled toys, and four novel toys not introduced to the dogs during the familiarisation. Two of these novel toys (novel A toys) were used in the first trial, and the other two (novel B toys) were used in the second trial. All the toys were washed with soap before the test to control for odour cues. Before the test, the caretaker entered the room without the dog and prepared the setup by placing the 6 toys on the floor: the two labelled, the two unlabelled and the first pair of novel toys (novel A toys), approximately 30 cm apart. They then stood 1.5 m away from the toys, let the dog in and ignored them by looking at their feet for the duration of the test. After the first trial, the dog had a 5-minute break during which it was not allowed in the testing room. The caretaker washed the two labelled, two unlabelled and two novel B toys. The second trial was then conducted in the same way, but the two novel A toys were replaced by the two novel B toys, which were different from the two novel A toys.

### Vocabulary assessment test

After the test described above, the acquisition of the labels of the labelled toys was evaluated through a Vocabulary Assessment Test (VAT, Dror et al. 2023). The purpose of this test was to confirm whether dogs had learned the names of the labelled toys and could reliably retrieve those on command. This test was conducted after the main experiment. We tested the dogs in eight trials, in which all eight toys (2 labelled, 2 unlabelled and 4 novel toys) were scattered on the floor about 30 cm apart. The caretaker was asked to instruct their dog to retrieve the two labelled toys, one at a time (4 trials for each toy), alternating between them in a semi-randomised order, with no more than two consecutive trials on the same toy. After every trial, the caretaker returned the toy fetched by the dog to the toy’s room, so the dog always had 8 toys to choose from. The caretaker’s view of the toys was blocked during the trials to ensure they did not inadvertently cue the dog, following the same procedure we used in the past (Fugazza et al. 2021). During the test, the Experimenter noted whether the dog fetched the requested object or a different one.

### Data collection

The tests were recorded remotely using an online software (i.e., Streamyard). The footage was coded using the behaviour-coding software BORIS v. 8.11.1 (Friard and Gamba 2016). Two coders independently observed the behaviour of the dogs in both trials using the following behavioural variables (Table 1).

**Table 1:** This table describes the main variables examined in the experiment

Variable	Description
Interaction (labelled toy)	The dog physically interacts with either labelled toy (i.e., chewing it, sniffing it). We quantified this behaviour as the duration of interaction, expressed as a proportion of the total trial time
Interaction (unlabelled toy)	The dog physically interacts with either unlabelled toy (i.e., chewing it, sniffing it). We quantified this behaviour as the duration of interaction, expressed as a proportion of the total trial time
Interaction (novel toy)	The dog physically interacts with either novel toy (i.e., chewing it, sniffing it). We quantified this behaviour as the duration of interaction, expressed as a proportion of the total trial time
Looking	The dog orients their gaze toward the caretaker while not in physical contact
Offering	The dog actively presents or “offers” a toy toward the caretaker (e.g., fetches a toy and places it near the caretaker’s feet, holds it in the mouth while facing the caretaker, or alternates gaze between the toy and the caretaker while close)
Contact	The dog maintains physical contact with the caretaker (touching any part of the caretaker’s body with its head or body) while not offering a toy
*Other*	Any behaviour not classified as Looking, Offering, or Contact (e.g., independent exploration of the room or toys)

### Data analysis

To estimate how the proportion of time spent interacting with a toy was influenced by the interaction between group and type of toy, and to analyse the proportion of time individuals spent interacting with their caretaker, we employed two types of mixed models.

For the analysis of toy interaction time, we fitted a Generalised Linear Mixed Model (GLMM; Baayen 2008) using a beta error distribution with a logit link function (Bolker 2008; McCullagh 2019). The model included group, type of toy, their interaction, and trial number as fixed effects. Dog ID was included as a random intercept to account for repeated measures. To control type I error rates and avoid overly confident estimates (Barr 2013; Schielzeth and Forstmeier 2009), we also incorporated random slopes for the type of toy and trial number within dog ID. Due to convergence issues, we employed an uncorrelated random slope structure, omitting correlation parameters between random slopes and intercepts. To test group effects and their interaction with toy category, we compared the full model to a reduced model excluding the interaction term. Because the response consisted of independent proportions bounded between 0 and 1, we used a beta-distributed GLMM, which requires all observations to fall strictly within the interval (0, 1). We therefore replaced exact boundary values following Smithson and Verkuilen (2006). For each trial, the proportion of time spent interacting with each toy type (novel, labelled, unlabelled) was calculated by dividing the interaction duration by the total available time (90 s). The trial number was z-transformed, thereby improving model convergence and facilitating the interpretation of coefficients (Schielzeth 2010). The type of toy was manually dummy-coded and centred before inclusion as a random slope. We fitted the model in R (version 4.4.2; R Core Team 2021) using the function glmmTMB from the glmmTMB package (version 1.1.10; Brooks et al. 2017). Significances were assessed using likelihood ratio tests (Dobson and Barnett 2018). Model stability was assessed by excluding individual dogs one at a time and comparing model estimates from the resulting subsets to those from the full dataset, revealing good stability. Confidence intervals for model estimates and fitted values were obtained via parametric bootstrapping (*N* = 1000) using the simulate function from the glmmTMB package. The dispersion parameter indicated no overdispersion (1.057), and collinearity was not an issue (maximum VIF = 1), assessed via a linear mixed model (LMM) excluding random effects. In this toy-interaction model, T dogs were used as the reference group.

To analyse how the proportion of time individuals spent engaging in four categories of behaviour (looking, offering, and contact, with a residual other category) was influenced by group and trial, we fitted a Linear Mixed-Effects Model (LMM; Baayen 2008). The behavioural response had a different data-generating structure from the toy-interaction analysis: the four proportions per trial were mutually exclusive and summed to 1, forming a compositional response. Because compositional data are constrained to a simplex and the components are inherently dependent, beta regression is not suitable for this type of response. Following standard compositional data analysis procedures, we first replaced exact zero values to allow log-ratio transformation (Smithson and Verkuilen 2006). Subsequently, for each trial and subject, we applied a centred log-ratio transformation (Van den Boogaart and Tolosana-Delgado 2013), projecting the data into an unconstrained space suitable for linear modelling. Model predictions were obtained in this space and back-transformed to the original, doubly constrained scale for interpretation. Fixed effects included group, behaviour, their interaction, trial number, and the interaction between trial number and behaviour. As each trial yielded four behavioural proportions, the group × behaviour interaction was the key term of interest, capturing whether the distribution of behaviours differed systematically across groups. Trial number was z-transformed to a mean of zero and a standard deviation of one to facilitate convergence and interpretation (Schielzeth 2010). Random intercepts were included for subject ID and trial (nested within subject). To account for repeated measures and avoid inflated type I error rates (Schielzeth and Forstmeier 2009; Barr 2013), random slopes for behaviour and trial number were also incorporated. Due to the limited number of observations per subject (four per trial), correlation parameters among random effects were not estimated. Behaviour was manually dummy-coded and centred before inclusion as a random slope. In this model, GWL dogs served as the reference group. The model was fitted in R using the function lmer from the lme4 package (version 1.1–35.5; Bates et al. 2015) with maximum likelihood estimation. To test the significance of the group × behaviour interaction, we refitted the model using restricted maximum likelihood (REML) and determined degrees of freedom using the Satterthwaite approximation (Luke 2017), implemented via the lmerTest package (version 3.1-3.1; Kuznetsova et al. 2017). Confidence intervals for parameter estimates and fitted values were derived via parametric bootstrapping (*N* = 1000) using custom extensions of lme4 functions. Model diagnostics indicated no violations of assumptions: inspection of QQ-plots, residuals vs. fitted plots, and random-effects distributions revealed normally distributed and homoscedastic residuals. Model stability was evaluated using a leave-one-out procedure implemented via custom stability functions, which confirmed good stability. Inspection of individual data points revealed some extreme values, but model diagnostics and stability analyses indicated that these observations did not exert undue influence on the estimates. As they reflect genuine individual variation rather than measurement artefacts, they were retained in all analyses.

The final dataset comprised 58 trials (each yielding four behavioural proportions and corresponding measures of time spent interacting with each toy type) conducted with 29 individuals. Inter-rater agreement was excellent for all variables, as assessed by Cronbach’s alpha (α > 0.90), which we used here as a measure of consistency across the two coders.

## Results

### Toy interaction

**Fig. 2:**
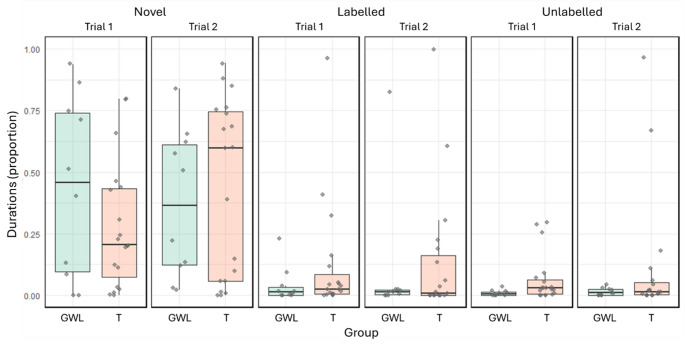
Box plots and jittered points show the proportion of time spent by dogs in different groups (GWL and T) interacting with different types of toys across trials. Each boxplot illustrates the distribution of data: the box represents the interquartile range (IQR), containing the middle 50% of the data, with the median indicated by a line inside the box. The whiskers extend to the smallest and largest values within 1.5 times the IQR from the quartiles

Overall, the group had no significant effect on the time spent interacting with toys (full–null model comparison, likelihood ratio test: χ² = 1.909, df = 3,*p* = 0.591). T dogs and GWL dogs interacted with both labelled and unlabelled toys for similar durations, and no significant group differences were observed when engaging with novel toys (Table 2). Notably, dogs from both groups showed a clear preference for novel toys, spending significantly more time interacting with them than with labelled or unlabelled toys (χ² = 28.784, df = 2,*p* < 0.001; Fig. 2).

**Table 2:** Results of the model examining the time spent interacting with each type of toy. Shown are fixed-effect estimates with their standard errors (SE), 95% confidence limits (CL), likelihood-ratio χ² tests, degrees of freedom (df), and p-values. Group was dummy-coded with T as the reference level, toy type was dummy-coded with labelled as the reference level, and trial number was z-transformed (mean = 1.50, SD = 0.50)

Term	Estimates	SE	CL_(lower)_	CL_(upper)_	χ^2^	df	*p*
Intercept	−1.799	0.257	−2.319	−1.275			
Group	0.218	0.303	−0.397	0.812			
Interacting w novel	1.253	0.355	0.570	2.024	28.784	2	0.001
Interacting w unlabelled	−0.165	0.344	−0.889	0.559			
Trial	0.037	0.084	−0.130	0.214			
GroupT x novel	−0.403	0.435	−1.328	0.437			
GroupT x unlabelled	0.087	0.427	−0.798	0.928			

### Caretaker-directed behaviour during toy interaction

**Fig. 3:**
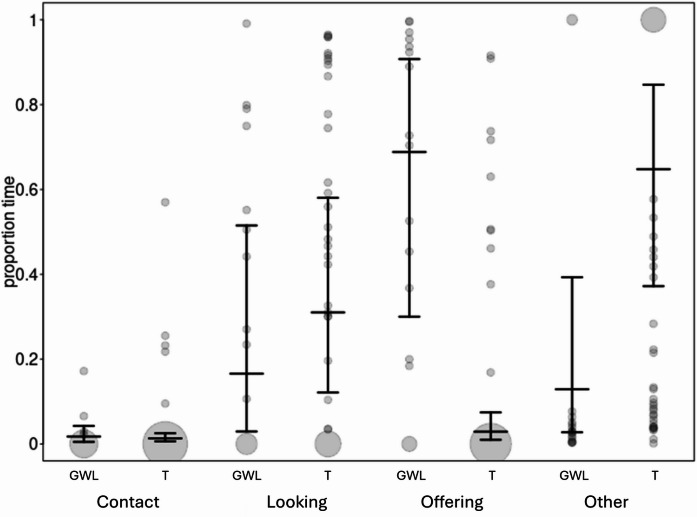
Proportion of time spent with different behaviours, separately for the T and GWL dogs. Dots represent the raw proportions recorded on each sampling day; darker shading indicates overlapping observations, and larger dot size reflects multiple identical values at the same position. Black bounded vertical lines show the fitted model estimates and their 95% confidence intervals

The full model differed significantly from the null model (likelihood ratio test: χ² = 17.126, df = 4, *p* = 0.002). The group × behaviour interaction was significant, indicating that the distribution of behaviours differed between groups (Table 3; Fig. 3). Compared with GWL dogs, T dogs spent significantly less time Offering (estimate = − 2.888, SE = 0.929, t = − 3.108, df = 30.4,*p* = 0.004) and more time in Contact with their caretakers (estimate = 1.894, SE = 0.837, t = 2.262, df = 29.8,*p* = 0.031). At the same time, no group difference was found for Looking (estimate = 0.906, SE = 0.987, t = 0.918, df = 29.1,*p* = 0.366). Trial number and its interaction with behaviour were not significant (all *p* > 0.42).

**Table 3:** Results of the compositional model. Indicated are estimates, together with their standard errors, 95% confidence limits, and significance tests. Behaviour was dummy coded with contact being the reference level, and group was dummy coded with G being the reference level. Trial was z-transformed to a mean of zero and a standard deviation of one; the mean and standard deviation of the original variable were 1.50 and 0.50. Tests for group × behaviour and trial × behaviour represent overall interaction effects

Term	Estimate	SE	CL_(lower)_	CL_(upper)_	t	df	*p*
(Intercept)	−1.986	0.420	−2.778	−1.217			
Group	0.022	0.506	−0.912	0.901			
Looking	2.257	0.820	0.728	3.782			
Offering	3.681	0.772	2.292	5.192			
Contact	2.006	0.695	0.741	3.303			
Trial	−0.077	0.183	−0.440	0.290			
Group x looking	0.906	0.987	−0.918	2.712			
Group x offering	−2.888	0.929	−4.595	−1.231	−3.108	30.413	0.004
Group x contact	1.894	0.837	0.318	3.465	2.262	29.826	0.031
Trial x looking	0.054	0.259	−0.497	0.555			
Trial x offering	0.047	0.259	−0.439	0.552			
Trial x contact	0.208	0.259	−0.280	0.689			

### Vocabulary assessment test

Only the GWL dogs selected the correct labelled toys above chance upon hearing their names (binomial test, all *p* < 0.001, Table 4), while the T dogs failed to do so (all *p* > 0.05).

**Table 4:** Choice accuracy of GWL dogs in the VAT. For each dog, the table reports the percentage of correct choices across all retrieval trials and the number of labelled toys available during testing. Dogs selected the correct toy from a set of eight toys; therefore, the chance level was 0.125 (1/8)

Dog ID	Type	Success rate	Toys on the floor
Binti	GWL	100%	8
Bryn	GWL	50%	8
Gani	GWL	50%	8
Lala	GWL	50%	8
Lexi	GWL	62.50%	8
Max	GWL	50%	8
Nalani	GWL	75%	8
Pippin	GWL	50%	8
Roy	GWL	100%	8
Squall	GWL	87.50%	8
Amy	T	0%	8
Ben	T	0%	8
Birka	T	0%	8
Dios	T	0%	8
Hailey	T	0%	8
Kiki	T	0%	8
Minka	T	0%	8
Mino	T	0%	8
Moki	T	0%	8
Naru	T	0%	8
Nola	T	0%	8
Poppy	T	0%	8
Roze	T	0%	8
Rumini	T	0%	8
Sidney	T	0%	8
Sintra	T	0%	8
Teo	T	0%	8
Vili	T	0%	8
Zia	T	0%	8

## Discussion

Our results provide the first evidence for differences between the behaviour of dogs with and without the ability to acquire a vocabulary of object names (GWL and T dogs, respectively) in the presence of novel and familiar (labelled and unlabelled) toys. Specifically, we did not find differences in their interactions with the familiar or novel toys. However, GWL dogs were more likely than T dogs to initiate interactions with their caretakers and, interestingly, were more likely to approach them with a novel toy in their mouth and actively initiate an interaction (see discussion below). In addition, all dogs interacted more with novel objects overall, confirming the neophilic attitudes of this species. Finally, the results of the VAT showed that only the GWL dogs, but not the T dogs, learned the labels of the two labelled toys during the familiarisation phase. This confirms the results of previous studies (Dror et al. 2023; Fugazza et al. 2021; Fugazza and Miklósi 2020) showing that only GWL dogs can acquire object verbal labels during playful interactions with their caretakers. Although a slight decrease in the number of toy interactions was observed from trial 1 to trial 2 across all dogs, this trend was not significant in the model. It may simply reflect a mild decline in motivation, which is expected given the caretaker’s passive role during the trials.

Taken together, the results of this study do not provide support for the presence of labelling biases or greater preference for novel toys in GWL dogs but raise the hypothesis that social motivation and tendency to seek social attention may be related to vocabulary acquisition in dogs.

Contrary to our expectations, we did not find an increased interest in labelled items in GWL dogs. This negative result could be due to several reasons, including the lack of existence of such an effect in dogs. It is also possible that the exposure to the toys was rather long, and such an effect may only emerge with shorter exposures, when the toys are novel, before the knowledge of the labels is consolidated through repetition. It is also possible that in our test, the presence of the novel toys masked the potential labelling effect by catalysing the interest of all the dogs almost exclusively in the novel toys. This novelty preference is in line with previous findings (Kaulfuß and Mills 2008; Kniowski 2012).

We also found no evidence that GWL dogs were more interested in the novel objects than typical dogs. Although toy motivation is not enough for label learning to emerge (Fugazza et al. 2021), it is still clearly essential for engaging dogs in the kind of playful interactions where label learning typically occurs (Dror et al. 2023; Fugazza et al. 2021). Therefore, meaningful comparisons between GWL and T dogs should be limited to individuals who are similarly motivated to play with toys. We expected GWL dogs to show more interest than T dogs in the novel toys, but instead, not only all dogs in the present study show comparable interest in familiar toys, but also they showed a comparably strong overall preference for the novel ones (Kaulfuß and Mills 2008; Kniowski 2012).

Interestingly, the results of our experiment suggest that GWL dogs may have a stronger motivation for social interactions involving objects than T dogs. Although T dogs spent slightly more overall time in passive contact with their caretakers, GWL dogs showed more active object-related behaviours such as Offering the toys, reflecting a difference in the style of social behaviour rather than in overall sociality. A potential explanation for the GWL dogs’ greater engagement could be their previous extensive experience playing with toys with their caretakers. However, when GWL dogs displayed Offering behaviour, they almost exclusively retrieved the novel toy, with no evidence of switching between toy types across trials. If the previous reinforcement history of playing with toys with their caretakers was the only decisive factor, they should have tended to bring the familiarised toys, which had a longer and more recent reinforcement history. Instead, it could be argued that their previous experience with their caretakers engaging in social playful interactions involving giving a name to novel toys may have played a role. Hence, it could be speculated that GWL dogs’ behaviour could be interpreted as an attempt to involve their caretakers in the social interaction surrounding the novel objects, possibly as a request to initiate play, including labelling. This behaviour appears to share some functional similarities with infants’ behaviour of pointing and alternating their gaze between the object and the caregiver (Carpenter et al. 1998), which, in the case of infants, is interpreted as aimed at sharing attention and initiating a communicative context. This interpretation raises the intriguing hypothesis that social motivation to share attention and initiate a communicative context may be at the basis of GWL dogs’ label learning skills. The higher social interest of GWL dogs in this situation is also consistent with previous research showing that GWL dogs are more playful than T dogs (Fugazza et al. 2022) and that they are social learners, acquiring toy names while playing with their caretakers (Dror et al. 2023; Fugazza et al. 2021). However, this fascinating hypothesis has to be taken with caution because other alternative explanations, such as more extensive experience of playing with the caretakers, still need to be eventually excluded. In fact, it is also possible that more playful caretakers unknowingly teach their dogs a specific way to interact with toys and that this playfulness, in turn, affects the dogs’ tendency to play and engage in social interactions involving toys.

We did not collect formal data on caretakers’ toy-play frequency at home. We recognise that unmeasured differences in daily play routines could partly explain the observed differences and should be documented in future research. However, caretakers provided consistent time estimates for the standardised familiarisation phase, and overall toy engagement during test phases was similar across groups.

Another limitation of this study is that our experiment was not designed to measure toy preferences per se; therefore, we cannot entirely exclude the possibility that pre-existing preferences subtly influenced behaviour. More targeted preference tests would help clarify this aspect. This study only included Border collies in both experimental groups. We have no specific reason to assume that the present results would apply only to Border collies; however, some caution should be taken in generalising the results in the absence of tests carried out on other breeds.

## Conclusions

Whereas we did not find support for the hypothesis of a labelling effect in GWL dogs, the results showing a higher tendency of GWL dogs, compared to typical dogs, to initiate social interactions with the caregivers lay the foundation for future studies to explore the relationship between social motivation, communicative intent and vocabulary learning in non-human and non-linguistic species. This approach is expected to provide a comparative framework to study the evolution of cognitive skills related to the acquisition of verbal labels.

## Data Availability

Raw data from the experiment is available upon request.
